# Enhancing Patient Comfort in Aesthetic BoNT-A Injections: Mechanical Versus Topical Analgesia

**DOI:** 10.1007/s00266-026-05884-2

**Published:** 2026-05-13

**Authors:** Emre Güvercin, Nilüfer Bahadırlı, Burak Ergün Tatar

**Affiliations:** 1 Department of Plastic, Reconstructive and Aesthetic Surgery, Faculty of Medicine, Maltepe University, Istanbul, Turkey; 2 Plastic, Reconstructive and Aesthetic Surgeon, Private Practice, Istanbul, Turkey; 3 Department of Plastic, Reconstructive and Aesthetic Surgery, Medipol University, Medipol Acıbadem District Hospital, Istanbul, Turkey

**Keywords:** Botulinum toxins, Type A, Pain management, Facial aesthetics, Topical anesthetics, Mechanical stimulation

## Abstract

**Introduction:**

Botulinum toxin type A (BoNT-A) is widely used in aesthetic medicine to treat dynamic facial rhytides. Despite its well-established safety and efficacy, BoNT-A injections often induce discomfort and anxiety owing to injection-related pain, especially in sensitive facial areas. Although topical anesthetic creams are commonly employed to alleviate pain, they have limitations, such as delayed onset and local side effects. Mechanical analgesic devices, based on the gate control theory of pain, have emerged as promising non-pharmacological alternatives.

**Methods:**

In this prospective, single-center, split-face study, 80 adult patients undergoing upper-facial BoNT-A injections between May 2024 and August 2025 were enrolled. Each participant received a topical anesthetic cream on one side of the face and a Shot Blocker-like mechanical analgesic device on the contralateral side. All injections were administered by a single experienced physician. Pain levels were assessed immediately post-injection using a visual analog scale (VAS, 0–10) by a blinded evaluator. Statistical analyses included paired t-tests, Wilcoxon signed-rank tests, Spearman correlation, and Mann–Whitney U tests. Effect sizes were reported using Cohen’s *dz* and Cliff’s Δ.

**Results:**

Mean VAS scores were significantly lower with the mechanical device (3.19 ± 1.08) than with the topical cream (4.14 ± 1.30) (*p* < 0.001). The intra-individual mean difference (0.95 ± 1.41) favored the mechanical device. Spearman’s correlation indicated that higher pain scores with the cream correlated with greater relative benefit from the device (ρ = 0.664, *p* < 0.001). Patients with higher baseline pain (VAS ≥ 4 on the cream side) experienced significantly greater pain reduction (*p* < 0.001).

**Discussion:**

A shot blocker-like mechanical analgesic device is more effective than a topical anesthetic cream in minimizing pain during BoNT-A injection into the upper face. Its immediate analgesic effect, absence of pharmacologic side effects, and ease of use make it a valuable adjunct in aesthetic practice, particularly for patients with pain sensitivity. Further studies are warranted to validate these findings in broader populations and different anatomical regions.

**Level of Evidence II:**

This journal requires that authors assign a level of evidence to each article. For a full description of these Evidence-Based Medicine ratings, please refer to the Table of Contents or the online Instructions to Authors.

**Supplementary Information:**

The online version contains supplementary material available at 10.1007/s00266-026-05884-2.

## Introduction

Botulinum toxin type A (BoNT-A) injections are among the most commonly performed aesthetic interventions worldwide, particularly for the management of dynamic facial rhytides. Although the procedure is widely regarded as safe and effective, pain associated with BoNT-A injections remains a frequently reported source of discomfort and anxiety among patients, especially when treating highly sensitive facial areas [1].

To alleviate injection-related pain, topical anesthetic creams, most commonly lidocaine/prilocaine formulations, are routinely applied prior to administration [2] . However, these agents have several drawbacks, including delayed onset, inconsistent dermal penetration, and the potential for adverse local reactions, such as erythema, irritation, or allergic responses [3]. These limitations have driven growing interest in alternative, non-pharmacological strategies for procedural pain management [4] .

One such approach involves the use of mechanical analgesic devices based on the gate control theory of pain. This theory suggests that non-noxious sensory input, such as pressure or vibration, can inhibit the transmission of nociceptive signals by stimulating large-diameter afferent nerve fibers, effectively "closing the gate" to pain perception [5].

The ShotBlocker® is a noninvasive, reusable device featuring a blunt plastic disk with multiple short projections. Designed to provide simultaneous cutaneous stimulation during needle-based procedures, it has demonstrated efficacy in reducing pain associated with subcutaneous and intramuscular injections in both pediatric and adult populations [6]. Recently, similar devices have been explored for their applicability in aesthetic procedures, including dermal fillers and mesotherapy injections.

Despite their promising utility, the analgesic effectiveness of mechanical stimulation devices has not yet been evaluated in the context of facial BoNT-A injections, a setting in which patients often present heightened sensitivity and elevated expectations regarding comfort. This study aimed to address this gap by comparing the analgesic efficacy of a ShotBlocker-like mechanical device with that of a standard topical anesthetic cream during upper facial BoNT-A injections. A prospective split-face design was employed to systematically assess patient-reported pain levels and determine the relative effectiveness of each modality.

## Materials and Methods

This prospective, single-center, split-face study was conducted between May 2024 and August 2025. Eighty adult patients who underwent botulinum toxin type A (BoNT-A) injections for the aesthetic treatment of the upper face were enrolled. All procedures were performed by the same experienced physician (E.G.) to ensure procedural standardization.

All procedures were performed as part of routine clinical aesthetic practice. Written informed consent was obtained from all participants before the procedure, including consent for the use of anonymized clinical data for research purposes. The study was conducted in accordance with the principles of the Declaration of Helsinki.

### Patient Selection

The inclusion criteria were adult patients seeking cosmetic botulinum toxin type A (BoNT-A) treatment for the glabellar, frontalis, and lateral canthal regions. The exclusion criteria were a history of facial surgery, neuromuscular disorders, active skin infections, chronic systemic diseases, pregnancy, and concurrent facial aesthetic interventions.

### Split-Face Study Design

A split-face design was applied to each participant. The side receiving topical anesthesia and the side receiving mechanical analgesia were alternated sequentially between patients to minimize allocation bias. To reduce potential order effects, the side injected first was also alternated between participants.

On one hemiface, a topical anesthetic cream containing lidocaine 2.5% and prilocaine 2.5% was applied as a thin uniform layer approximately 20 min prior to injection. No occlusive dressing was used. Immediately before injection, the cream was removed using sterile gauze, and the skin was cleansed with chlorhexidine antiseptic solution.

A mechanical analgesic device resembling a ShotBlocker® was used on the contralateral hemiface. This fenestrated plastic device was positioned over the injection site such that the central opening allowed needle entry, and the surrounding blunt projections provided cutaneous mechanical stimulation. The device was gently applied to the skin surface to provide neurosensory stimulation based on the Gate Control Theory of Pain without altering the injection technique or needle placement. The device was disinfected between patients using standard antiseptic solutions.

### Injection Technique

**Fig. 1:**
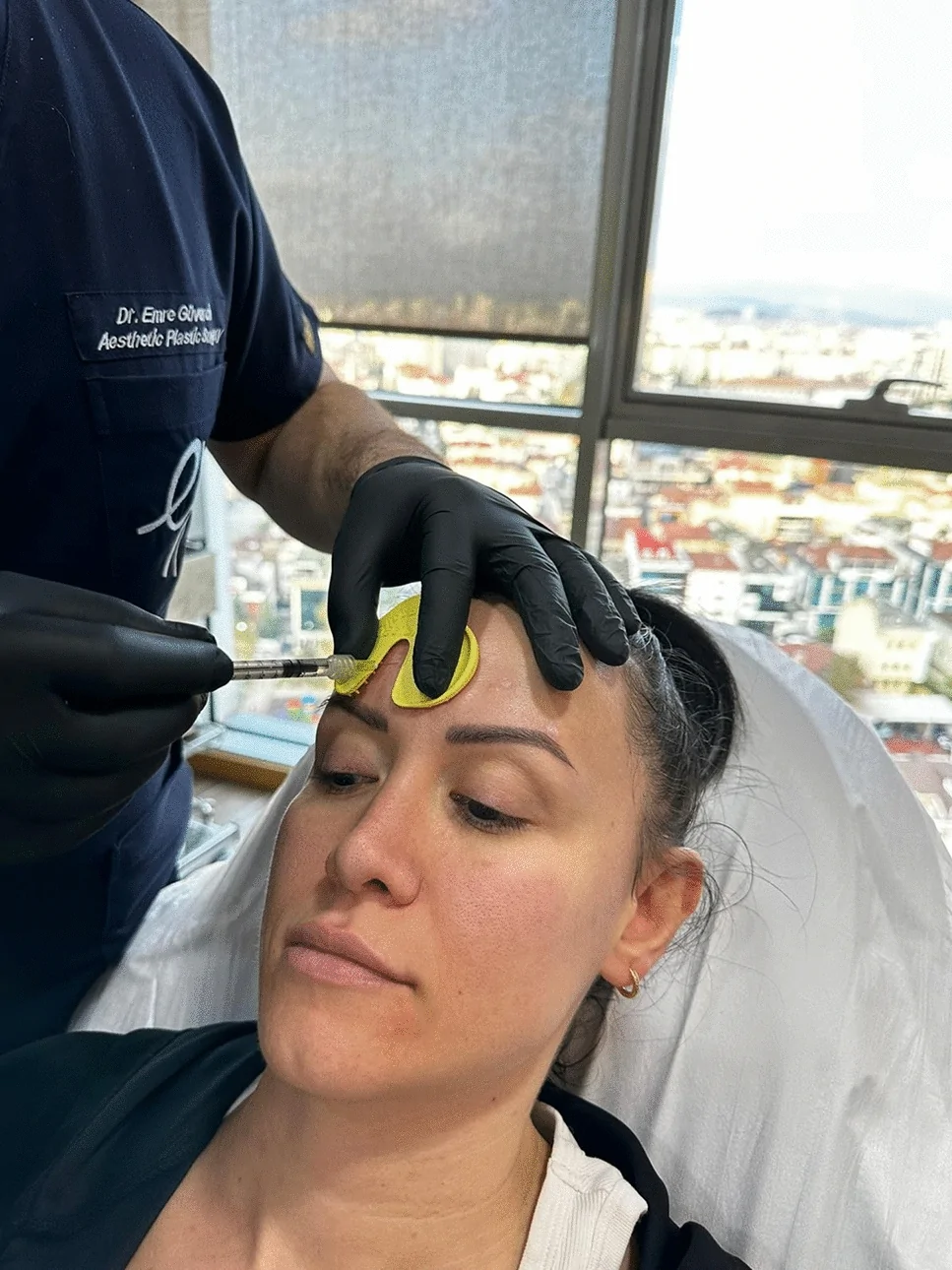
Application of botulinum toxin type A (BoNT-A) injections to the glabellar and forehead regions using a ShotBlocker-like mechanical analgesic device. The fenestrated device was positioned with the window aligned proximally to allow direct visualization of the injection site

**Fig. 2:**
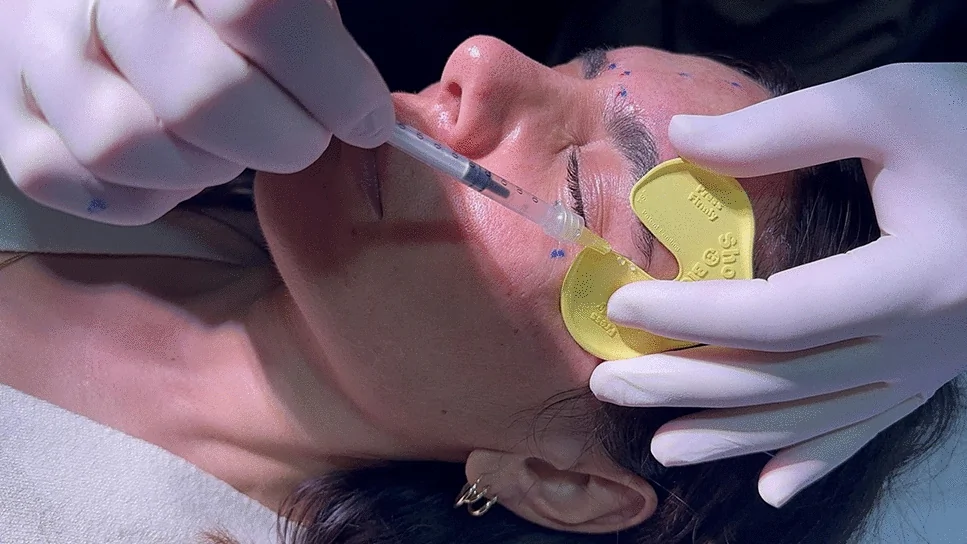
Use of a shotBlocker-like mechanical analgesic device during BoNT-A injection targeting the lateral canthal lines (crow’s feet). The device projections were oriented along the dermatomal distribution to maximize the tactile stimulation and pain modulation

After antiseptic preparation, standard injection points for upper facial BoNT-A treatment—including the glabellar, frontalis, and lateral canthal regions—were marked using a sterile surgical marker (Figs. 1-2, Video 1).

All injections were performed using Dysport^®^ (Ipsen Biopharm Ltd., UK), reconstituted with 2.5 mL of sterile saline to achieve a standardized dilution. A 30-gauge insulin syringe was used for all injections.

Because the procerus injection is located at the midline, this point was excluded from the side-specific pain comparison to maintain the validity of the split-face analysis.

### Pain Assessment

Pain intensity was assessed using a visual analog scale (VAS) ranging from 0 (no pain) to 10 (worst imaginable pain). Patients were asked to self-report their pain immediately after each injection by marking the VAS scale. Pain scores were recorded before cold compression.

### Post-Procedure Care

After completion of the injections and VAS assessment, bilateral cold compression was applied to reduce postprocedural discomfort and swelling.

### Statistical Analysis

All statistical analyses were performed using IBM SPSS Statistics for Windows, version 27.0 (IBM Corp., Armonk, NY, USA). Because of the split-face design, each participant served as their own control.

VAS scores between the topical anesthetic cream and mechanical device sides were compared using paired statistical tests. Descriptive statistics are presented as mean ± standard deviation (SD) and median with interquartile range (IQR). Depending on the normality of the distribution, either the paired t-test or Wilcoxon signed-rank test was used.

Spearman’s rank correlation analysis was performed to evaluate the relationship between baseline pain scores on the cream side and the intra-individual difference between treatments (cream—device). Participants were categorized into high- and low-pain groups based on the median VAS score of the cream side. These exploratory subgroup analyses should be interpreted cautiously because of potential statistical limitations.

Effect sizes are reported as Cohen’s dz for parametric paired comparisons and Cliff’s delta for nonparametric analyses. All statistical tests were two-tailed, and p < 0.05 was considered statistically significant.

## Results

Eighty patients who underwent BoNT-A injections in the upper face were included in the analysis. The mean age of the participants was 33.2 years. Of the cohort, 70 (87.5%) were women and 10 (12.5%) were men.

Pain intensity was assessed using a visual analog scale (VAS) ranging from 0 (no pain) to 10 (worst pain). On the side where the mechanical analgesic device was used, the mean VAS score was 3.19 ± 1.08, with a median of 3 (IQR: 2–4). On the side treated with the topical anesthetic cream, the mean VAS score was 4.14 ± 1.30, with a median of 4 (IQR: 3–5). The within-subject difference (cream—device) yielded a mean of 0.95 ± 1.41 and a median of 1 (IQR: 0–2), favoring the mechanical device (Tables 1-2).

**Table 1:** Comparison of pain intensity between topical anesthetic cream and mechanical analgesic device during upper facial BoNT-A injections

Variable	Cream side	Mechanical device side	Within-subject difference	*p*-value
Mean VAS (± SD)	4.14 ± 1.30	3.19 ± 1.08	0.95 ± 1.41	< 0.001 (*)
Median VAS (IQR)	4 (3–5)	3 (2–4)	1 (0–2)	< 0.001 (*)
Lower VAS on device side (%)	–	–	65.0%	–
Equal VAS on both sides (%)	–	–	18.8%	–
Higher VAS on device side (%)	–	–	16.2%	–
Effect size (Cohen’s dz)	–	–	0.67	–
Effect size (Cliff’s delta)	–	–	≈ 0.60	–

^*^Paired t-test and Wilcoxon signed-rank test used. Statistically significant at p < 0.05.

**Table 2:** Subgroup analysis of pain reduction according to baseline cream-side VAS scores

Variable	Low pain group (Cream-side VAS < 4)	High pain group (Cream-side VAS ≥ 4)	*p*-value
Number of patients (n)	40	40	–
Cream-side VAS (mean ± SD)	3.12 ± 0.61	5.16 ± 0.84	< 0.001
Device-side VAS (mean ± SD)	3.40 ± 0.92	3.65 ± 1.01	0.18
Within-subject difference (Cream—device, mean)	− 0.28	1.51	< 0.001
Median difference (IQR)	0 (− 1 to 1)	2 (1–3)	< 0.001
Patients benefiting from device (%)	37.5%	92.5%	< 0.001
Effect size (Cliff’s delta)	− 0.12	0.74	–

Statistical test: Mann–Whitney U test

Significance threshold: *p* < 0.05

A paired-sample analysis revealed a statistically significant reduction in pain with the mechanical device: t = 6.01, p < 0.001 (paired t-test) and W = 1812.5, p < 0.001 (Wilcoxon signed-rank test). Effect sizes indicated a moderate-to-large effect: Cohen’s dz = 0.67 and Cliff’s delta ≈ 0.60. Pain scores were lower on the mechanical device side in 65.0% of patients, equal in 18.8%, and higher in 16.2% of patients (Tables 1-2).

**Fig. 3:**
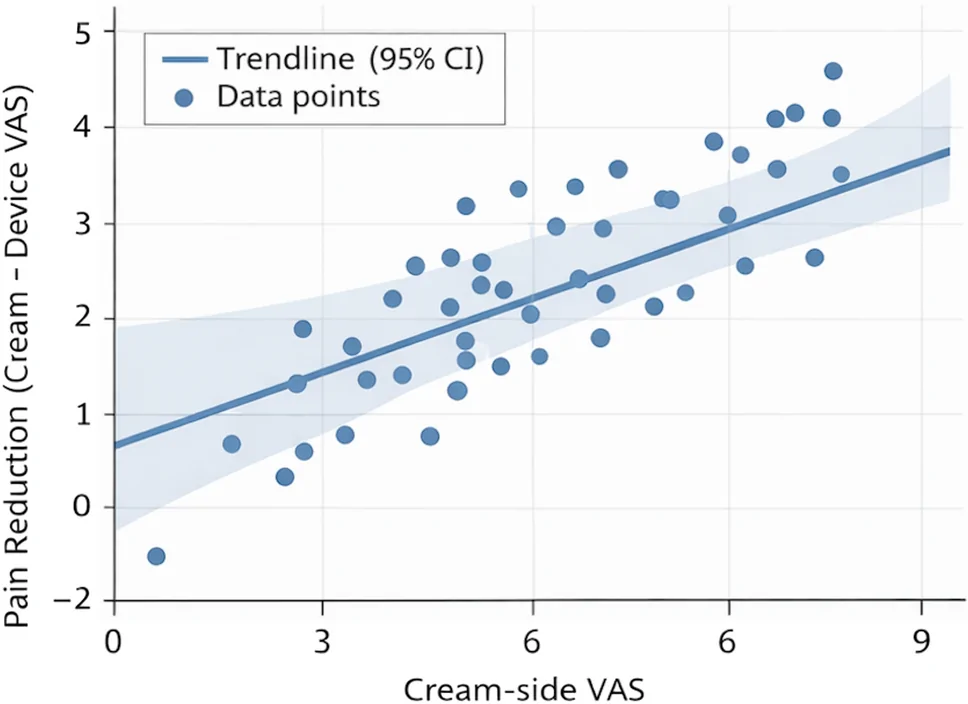
Scatter plot showing the correlation between baseline pain scores under the topical anesthetic cream condition (Cream-side VAS) and the magnitude of pain reduction achieved with the mechanical device (cream—Device VAS). A significant positive correlation was observed (ρ = 0.664, *p* < 0.001), indicating that patients who experienced higher baseline pain with the cream derived greater benefit from mechanical analgesic intervention. The shaded area represents a 95% confidence interval

A strong positive correlation was observed between the cream-side VAS score and the within-subject difference (ρ = 0.664, p < 0.001), suggesting that individuals experiencing more pain with the cream alone derived greater benefit from the mechanical device (Fig. 3).

**Fig. 4:**
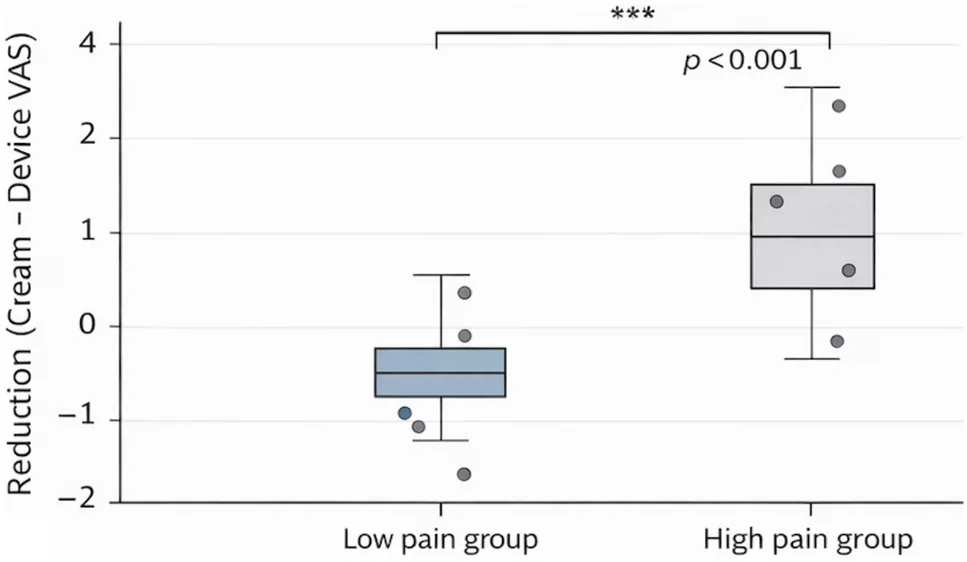
Boxplot comparison of within-subject pain reduction scores (cream—device VAS) between patients with low (VAS < 4) and high (VAS ≥ 4) baseline pain under the cream condition. Patients in the high-pain group experienced significantly greater analgesic benefit from the mechanical device (*p* < 0.001, Mann–Whitney U test), highlighting its enhanced efficacy in individuals with higher pain sensitivity

Patients were stratified based on the median cream-side VAS score (4) into high- and low-pain groups. The high-pain group showed a mean difference of 1.51, whereas the low-pain group showed a mean difference of −0.28. The between-group difference was statistically significant (U = 1166, p < 0.001, Mann–Whitney U test) (Fig. 4).

## Discussion

Our findings indicate that the use of a ShotBlocker-like compressive device was associated with lower pain scores during facial botulinum toxin (BoNT-A) injections than with the topical anesthetic cream protocol used in this study. These findings are consistent with previous studies that have demonstrated the analgesic potential of mechanical sensory modulation during injection-based procedures, including intradermal, intramuscular, and subcutaneous injections.

The observed analgesic effect may be explained by the Gate Control Theory of Pain, which proposes that non-noxious mechanical stimuli—such as the projections of the ShotBlocker device—activate large-diameter Aβ fibers that inhibit the transmission of nociceptive signals carried by smaller Aδ and C fibers, thereby reducing pain perception at the spinal or trigeminal level [7]. This mechanism is particularly relevant in facial procedures, where the trigeminal nerve branches (V1, V2, and V3) provide dense sensory innervation and patients may be more sensitive to discomfort during aesthetic injections [8].

Our results are in line with those reported by Yu et al., who demonstrated significantly reduced visual analog scale (VAS) pain scores during intradermal lidocaine injections when a compressive disc device was used. In their study, 93% of patients in the intervention group reported mild pain (VAS <30 mm) compared with 39% in the control group [9]. Similarly, Kuwahara and Ogawa reported that vibratory anesthesia reduced perceived pain during facial procedures, with 88% of participants reporting subjective improvement in comfort [8]. Unlike previous studies that primarily evaluated intradermal injections or vibratory analgesic devices, the present study examined a mechanical pressure-based analgesic tool during facial BoNT-A injections, a clinical context in which both injection depth and patient comfort are important considerations. In this regard, Gordin et al. reported that subcutaneous BoNT-A injections were perceived as less painful than intramuscular injections while maintaining therapeutic effectiveness [10]. These findings highlight the importance of procedural pain modulation strategies in aesthetic practice.

In the present study, the reduction in pain scores associated with the mechanical device appeared to be more pronounced among individuals who reported higher pain levels on the topical anesthetic side. A positive correlation was observed between cream-side VAS scores and the magnitude of pain reduction with the mechanical device, suggesting that mechanical sensory modulation may provide greater benefit in patients with higher baseline pain sensitivity or an inadequate response to topical anesthetics.

Similar observations have been reported in studies evaluating mechanical pain-reducing devices during invasive procedures [11]. Baxter et al. demonstrated that the use of the ShotBlocker during injections in pediatric patients resulted in significantly lower pain scores compared with standard injection techniques [12]. In our study, a similar trend was observed, particularly in patients for whom topical anesthetic cream alone did not provide sufficient analgesia.From a clinical standpoint, mechanical stimulation devices may offer several practical advantages compared with topical anesthetics. These include an immediate onset of analgesic effect, absence of pharmacological side effects, ease of application, and potential cost-effectiveness. However, ergonomic considerations, such as device positioning, adaptation to facial contours, and integration into routine injection workflows, should be taken into account to ensure consistent and reproducible application.

Nevertheless, several limitations should be acknowledged. Although the split-face design reduces interindividual variability, patient blinding was not possible, and expectation-related factors may have influenced pain perception. Additionally, the topical anesthetic cream was applied without occlusion and for a relatively short duration, which may have limited its analgesic effectiveness. Pain perception was evaluated using a subjective scale immediately after injection; therefore, psychological factors, such as anxiety or attentional bias, may have influenced reported scores. Finally, although the difference in VAS scores reached statistical significance, the clinical relevance of a mean difference of approximately one point on a ten-point scale should be interpreted with caution.

Future studies with larger patient populations and improved methodological controls, including randomized side allocation and region-specific pain assessment, may further clarify the role of mechanical sensory modulation during aesthetic injections. Comparative investigations examining different mechanical analgesia modalities, such as compression versus vibration, may also help determine the most effective strategies for procedural pain reduction.

In conclusion, within the limitations of this single-center split-face study, the use of a ShotBlocker-like mechanical stimulation device was associated with lower immediate pain scores during facial BoNT-A injections than with the topical anesthetic protocol. These findings suggest that mechanical sensory modulation may represent a useful adjunctive approach for improving patient comfort during aesthetic procedures.

## Supplementary Information

Below is the link to the electronic supplementary material.

Video 1: Demonstration of BoNT-A injection into the upper face using a ShotBlocker-like mechanical analgesic device. The video shows the application to the glabellar, frontalis, and lateral canthal regions. The fenestrated design allows direct visualization of the injection site while providing simultaneous mechanical stimulation along the dermatomal lines. All procedures were performed using standard dilution and injection techniques.

## References

[1] Salti G, Ghersetich I. Advanced botulinum toxin techniques against wrinkles in the upper face. Clin Dermatol. 2008;26(2):182–91. 18472059 10.1016/j.clindermatol.2007.09.008

[2] Yuen VH, Dolman PJ. Comparison of the three modified lidocaine solutions used in eyelid anesthesia. Ophthalmic Plast Reconstr Surg. 1999;15(2):143. 10189646 10.1097/00002341-199903000-00017

[3] Willey A, Lee PK. Cutaneous anesthesia. In: Roenigk RK, Roenigk HH, editors. Roenigk's dermatologic surgery: current techniques in procedural dermatology. 3rd ed. CRC Press; 2007. pp. 55–59.

[4] Drago LA, Singh SB, Douglass-Bright A, Yiadom MY, Baumann BM. Efficacy of ShotBlocker in reducing pediatric pain associated with intramuscular injections. Am J Emerg Med. 2009;27(5):536–43. 19497458 10.1016/j.ajem.2008.04.011

[5] Celik N, Khorshid L. The use of ShotBlocker for reducing pain and anxiety associated with intramuscular injection: a randomized, placebo-controlled study. Holist Nurs Pract. 2015;29(5):261–71. 26263287 10.1097/HNP.0000000000000105

[6] Caglar S, Büyükyılmaz F, Coşansu G, Çağlayan S. Effectiveness of ShotBlocker for immunization pain in full-term neonates: a randomized controlled trial. J Perinat Neonatal Nurs. 2017;31(2):166–71. 28437308 10.1097/JPN.0000000000000256

[7] Melzack R, Wall PD. Pain mechanisms: a new theory. Surv Anesthesiol. 1967;11(2):89–90.

[8] Kuwahara H, Ogawa R. Using a vibration device to ease pain during facial needling and injection. Eplasty. 2016;16:e9. 26933468 PMC4750366

[9] Yu J, Chen W, Liu Q, Mi J. Investigating 3D-printed disk compressing against skin for pain relief in intradermal infiltration anesthesia: a randomized controlled trial. BMC Anesthesiol. 2023;23(1):144. 37118673 10.1186/s12871-023-02088-y PMC10148480

[10] Gordin EA, Luginbuhl AL, Ortlip T, Heffelfinger RN, Krein H. Subcutaneous vs intramuscular botulinum toxin: split-face randomized study. JAMA Facial Plast Surg. 2014;16(3):193–8. 24699554 10.1001/jamafacial.2013.2458

[11] Uman L. Psychological interventions for needle‐related procedural pain and distress in children and adolescents. Cochrane Database Syst Rev. 2006;4:63. 10.1002/14651858.CD005179.pub2 17054243

[12] Baxter AL, Cohen LL, McElvery HL, Lawson ML, Von Baeyer CL. Integration of vibration and cold therapy relieves venipuncture pain in a pediatric emergency department. Pediatr Emerg Care. 2011;27(12):1151–6. 22134226 10.1097/PEC.0b013e318237ace4

